# Mitochondrion-targeted platinum complexes suppressing lung cancer through multiple pathways involving energy metabolism†
†Electronic supplementary information (ESI) available: NMR, ESI-MS, and HPLC; IC_50_, body weight of mice, biodistrubution of OPT, agarose gel electrophoresis, OCR of muscle mitochondria, fluorescence and TEM images of mitochondria, flow cytometric plot, and synthetic and experimental details. See DOI: 10.1039/c8sc04871a


**DOI:** 10.1039/c8sc04871a

**Published:** 2019-01-22

**Authors:** Zhenzhu Zhu, Zenghui Wang, Changli Zhang, Yanjun Wang, Hongmei Zhang, Zhenji Gan, Zijian Guo, Xiaoyong Wang

**Affiliations:** a State Key Laboratory of Pharmaceutical Biotechnology , School of Life Sciences , Nanjing University , Nanjing , P. R. China . Email: boxwxy@nju.edu.cn ; Fax: +86 25 83314502 ; Tel: +86 25 89684549; b State Key Laboratory of Coordination Chemistry , School of Chemistry and Chemical Engineering , Nanjing University , Nanjing , P. R. China . Email: zguo@nju.edu.cn ; Fax: +86 25 83314502 ; Tel: +86 25 89689006; c School of Biochemical and Environmental Engineering , Nanjing Xiaozhuang University , Nanjing , P. R. China; d State Key Laboratory of Pharmaceutical Biotechnology , Model Animal Research Center of Nanjing University , Nanjing , P. R. China

## Abstract

Mitochondria are potential therapeutic targets for anticancer drugs. A series of mitochondrion-targeted monofunctional platinum complexes, [Pt(*ortho*-PPh_3_CH_2_Py)(NH_3_)_2_Cl](NO_3_)_2_ (OPT), [Pt(*meta*-PPh_3_CH_2_Py)(NH_3_)_2_Cl](NO_3_)_2_ (MPT), and [Pt(*para*-PPh_3_CH_2_Py)(NH_3_)_2_Cl](NO_3_)_2_ (PPT) (PPh_3_ = triphenylphosphonium, Py = pyridine), are studied in this article. The antitumor activity and mechanism of action have been investigated *in vitro* and *in vivo* as well as on molecular levels. OPT exhibits higher efficacy than cisplatin against A549 lung cancer cells; furthermore, it shows a strong inhibition towards the growth of non-small-cell lung cancer in nude mice. The DNA binding ability of these complexes follows an order of PPT > OPT > MPT. Cellular uptake and distribution studies show that OPT accumulates mainly in mitochondria, while MPT and PPT accumulate more preferentially in nuclei than in mitochondria. As a result, OPT induces remarkable changes in the ultrastructure and membrane of mitochondria, leading to more radical mitochondrial dysfunctions than cisplatin. The release of cytochrome c from mitochondria is more evident for cells treated with OPT than with cisplatin, though the apoptosis of A549 cells induced by OPT is similar to that induced by cisplatin. Disruption to mitochondrial oxidative phosphorylation and glycolysis is involved in the antitumor mechanism of these compounds. The results indicate that in addition to DNA binding, bioenergetic pathways also play crucial roles in the antitumor activity of mitochondrion-targeted monofunctional platinum complexes.

## Abstract 2

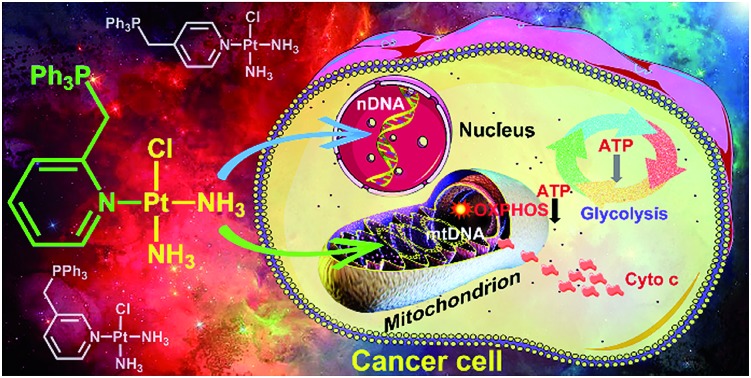
Monofunctional platinum complexes restrain lung cancer through disrupting mitochondrial oxidative phosphorylation and glycolysis in addition to damaging nuclear and mitochondrial DNA.

## Introduction

Lung cancer, particularly non-small-cell lung cancer (NSCLC), is the most common lethal disease, accounting for *ca.* 85% of all lung cancer cases.^1^ Patients with NSCLC only have a 10–15% 1 year survival rate even under the best treatment.^2^ Platinum-based anticancer drugs are the major chemotherapeutics used for treating various cancers,^3^–^5^ including NSCLC.^6^–^8^ These drugs are believed to react with nuclear DNA (nDNA) and induce apoptosis by inhibiting nDNA replication and gene transcription.^9^ However, their clinical efficacy is severely undermined by drug resistance,^10^ which results primarily from the decrease of cellular drug accumulation and increase of cellular self-repairation.^11^ Recent studies suggested that platinum drugs could interact with sub-cellular compartments other than nuclei.^9^

Mitochondria play central roles in cellular energy conversion, metabolism and apoptosis.^12^ Any mitochondrial abnormality such as respiration injury or membrane depolarization can be regarded as mitochondrial dysfunction.^13^ Energy metabolism in mitochondria is particularly important for balancing the energy requirements of cell maintenance.^14^ Mitochondria generate energy mainly through the oxidative phosphorylation (OXPHOS) pathway accompanied by the consumption of oxygen. In cancer cells, the hypoxic microenvironment limits the mitochondrial OXPHOS to generate ATP, and forces the cells to increase glucose glycolysis to compensate for the energy deficiency,^15^ thereby leading to the “Warburg effect”,^16^ a phenomenon that does not appear in normal differentiated cells. Although aerobic glycolysis is an inefficient way to generate ATP, it is a compensatory approach for the energy need of tumor growth.^17^ Therefore, direct interaction with mitochondria seems to be related to the drug-induced cell death,^18^ and complexes with mitochondrion-targeting properties may bring about a breakthrough in the design of platinum-based anticancer drugs.^19^–^21^ For instance, a Pt^II^ complex (mtPt) with a mitochondrial-penetrating peptide was delivered to mitochondria of human cancer cells, inducing apoptosis by damaging mitochondrial DNA (mtDNA) rather than nDNA,^22^ which suggests that mtDNA is a potential target for platinum-based anticancer drugs.

Previous reports suggested that compounds with delocalized positive charge and high lipophilicity could penetrate the inner mitochondrial membrane easily due to its high impermeability.^23^ Triphenylphosphonium (Ph_3_P^+^, TPP) is such a cation and able to accumulate in mitochondria due to the negatively charged microenvironment within the mitochondrial matrix.^24^ For example, platin-M, a Pt^IV^ prodrug of cisplatin with Ph_3_P^+^ as the axial ligand, was fabricated into a biocompatible polymeric nanoparticle for mitochondrion-targeted drug delivery, which can attack mtDNA and inhibit the cisplatin-resistant cancers.^25^ Recently, we demonstrated that two mitochondrion-targeted Pt^IV^ complexes bearing Ph_3_P^+^ as a homing moiety could significantly affect the mitochondrial bioenergetics of cancer cells and the cytotoxicity of the complexes.^26^

**Fig. 1:**
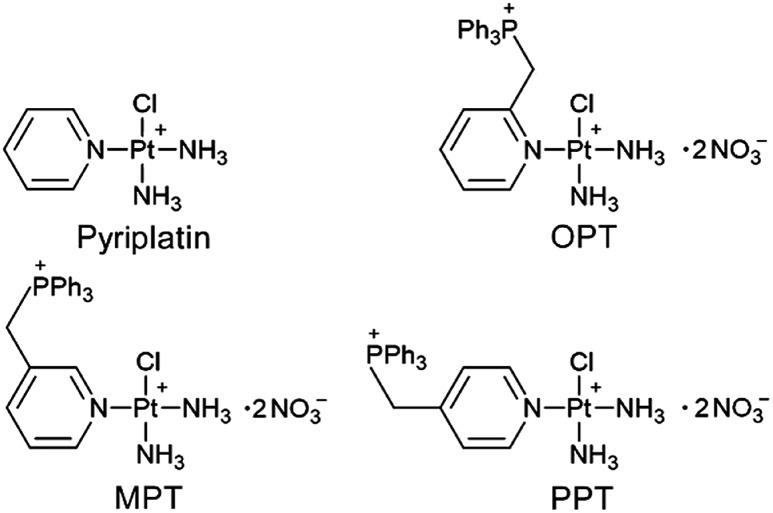
Structures of pyriplatin, OPT, MPT and PPT.

It was reported that the monofunctional Pt^II^ complex pyriplatin and its analogue phenanthriplatin could bind to DNA in a monodentate manner at the N7 position of guanine residues with no significant distortion of the double helix,^27^,^28^ which is different from cisplatin. In this study, we designed three monofunctional mitochondrion-targeted Pt^II^ complexes OPT, MPT and PPT by modifying pyriplatin with –CH_2_Ph_3_P^+^ (Fig. 1). The resulting complexes were fully characterized and evaluated for antitumor activity. Among them, OPT exhibited greater efficacy than cisplatin in A549 lung cancer cell lines and animal models with non-small-cell lung cancer. The cellular and mitochondrial accumulation, mitochondrial function and DNA binding ability of OPT were investigated. The results provide some new insights into the anticancer mechanism of platinum complexes in cancer cells.

## Results and discussion

### Synthesis and characterization

The synthesis of OPT, MPT and PPT was described in the ESI and Scheme S1.† The complexes were characterized by ^1^H-, ^31^P- and ^195^Pt-NMR, ESIMS and HPLC (Fig. S1–S5†). ESIMS results showed that the complexes in aqueous solution exist basically in an ionized form with two positive charges. These complexes possess delocalized positive charge and high lipophilicity, which may facilitate them to enter into cells easily through the lipid bilayer of the cellular membrane and further accumulate in the mitochondrial membrane matrix for the negative inside mitochondrial membrane potential (MMP, Δ*Ψ*_m_, *ca.* –180 mV).

### Antitumor activity

**Fig. 2:**
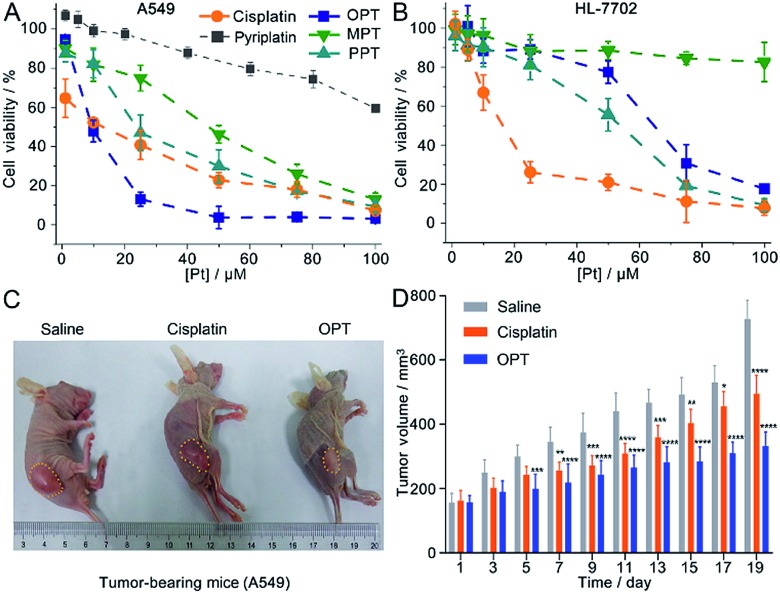
Cytotoxic profiles of the complexes against A549 (A) and HL-7702 cells (B) at 48 h, A549 tumor progression of mice treated with OPT (5 mg kg^–1^) (C), and changes of tumor volume after 19 d treatment (D). Cytotoxicity is presented as the mean ± S.D. of three independent experiments; tumor volume is presented as the average volume ± S.D. of 5 mice, *p < 0.1, **p < 0.01, ***p < 0.001, and ****p < 0.0001. Cisplatin and saline are used as controls.

The *in vitro* cytotoxicity of the complexes was investigated by the MTT assay against the human non-small-cell lung cancer A549, human cervical cancer HeLa, human liver cancer SMMC and human normal liver HL-7702 cell lines. The half maximal inhibitory concentration (IC_50_) of OPT against A549 cells (8.7 ± 1.6 μM) is the lowest (Table S1†), in that OPT inhibits cells more effectively as compared to MPT, PPT, cisplatin or pyriplatin (Fig. 2A). Under the same conditions, the IC_50_ values of OPT and PPT towards normal HL-7702 cells are 64.5 ± 3.2 and 53.7 ± 2.9 μM, respectively, which are much higher than that of cisplatin (14.8 ± 2.3 μM); MPT is almost nontoxic (Fig. 2B).

Since OPT exhibited the strongest cytotoxicity against A549 cells, mice implanted with A549 cells were used as xenograft models to evaluate the tumor inhibition efficacy of OPT. The mice were divided into 3 groups randomly and intravenously injected with saline, cisplatin and OPT, respectively, every other day for 19 days. Strong inhibition of tumor growth was observed after the treatment. On day 19, the tumor volume of the saline-treated mice was increased by *ca.* 5-fold as compared with that on the first day, whereas the average tumor volume of the OPT-treated mice grew much slower than that treated with cisplatin or saline (Fig. 2C and D). The body weight of the saline-treated groups was almost unchanged within 19 days, while that of the OPT-treated group decreased gradually with time, even more evident than that induced by cisplatin, which may result from the mitochondrion-disrupting effect of OPT (Fig. S6†). The biodistribution of Pt in major organs of mice was also determined. Most of the Pt was accumulated in the liver and kidneys (Fig. S7†), implying that the cellular uptake of OPT may be mediated by organic cation transporters (OCT1/2/3), which are primarily expressed in the liver and kidneys.^27^,^29^ Compared with cisplatin, more Pt was accumulated in the lungs and tumor tissue.

### Cellular and mitochondrial uptake

Due to the significant *in vitro* and *in vivo* antitumor effects, we further investigated the cellular uptake and distribution of the complexes. The content of Pt in mitochondria, nuclei, and mitochondrion-free cytoplasm separated from A549 cells after incubation with the complexes respectively is listed in Table 1. As expected, part of OPT, MPT and PPT can penetrate mitochondria and accumulate in the mitochondrial matrix. Cisplatin mainly accumulated in the nuclei, with only a limited amount of Pt being detected inside mitochondria, which is consistent with a previous report.^30^

**Table 1:** The content of Pt (μg L^–1^ per 10^6^ cells) in mitochondria (M), nuclei (N) and mitochondrion-free cytoplasm of A549 cells after incubation with OPT, MPT, PPT and cisplatin (10 μM), respectively, for 24 h

Organelles	OPT	MPT	PPT	Cisplatin
Mitochondria	8.18 ± 0.14	2.61 ± 0.02	2.40 ± 0.02	0.70 ± 0.01
Nuclei	4.06 ± 0.04	4.74 ± 0.03	10.07 ± 0.10	6.51 ± 0.04
Cytoplasm	2.79 ± 0.01	1.62 ± 0.02	1.32 ± 0.02	3.31 ± 0.05
Total	19.53 ± 0.58	9.90 ± 0.56	14.63 ± 0.23	12.46 ± 0.14
M/N	2.0	0.6	0.2	0.1

### Interaction with DNA

**Fig. 3:**
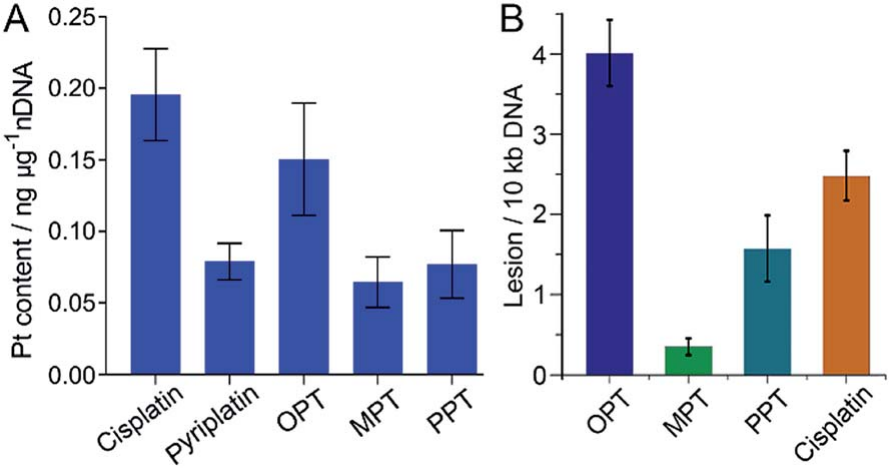
Content of nDNA-bound Pt in A549 cells after incubation with OPT, MPT, PPT, pyriplatin and cisplatin (10 μM), respectively, for 24 h (A), and damage to the D-loop region of mtDNA in A549 cells caused by OPT, MPT, PPT and cisplatin, respectively (B). Data are expressed as mean ± S.D. of at least three independent experiments.

It is widely accepted that nDNA is the main target for platinum-based anticancer drugs, and meanwhile mtDNA damage is sufficient to induce apoptosis of tumor cells.^31^–^33^ Therefore, we explored the interaction of the complexes with nDNA and mtDNA. Firstly, A549 cells were treated with OPT, MPT, PPT, pyriplatin and cisplatin, respectively, for 24 h. Nuclei were isolated from the cells and nDNA was obtained. The nDNA-bound Pt was measured by ICP-MS. As shown in Fig. 3A, the binding ability of OPT to nDNA is only second to that of cisplatin, much higher than that of other complexes. To evaluate the mtDNA lesion induced by the complexes, real-time polymerase chain reaction (PCR) assay was performed using four key regions of mitochondrial genome. Two mtDNA fragments of different lengths ranging from 972 to 1037 bp for long fragments and from 54 to 87 bp for short fragments located in the same mitochondrial genomic region were used as primers.^34^ One of the mitochondrial genomic regions is the D-loop, which exhibits a triple-stranded and semi-stable structure during replication. Due to its partially relaxed structure, the D-loop is more fragile than other mtDNA regions. The calculated frequency of the mitochondrial lesion in the D-loop region is displayed in Fig. 3B. OPT induced the most prominent mtDNA lesion among the three complexes and cisplatin, while all the complexes showed no effect on the other three mitochondrial genomic regions.

To further understand the relative DNA-binding ability of the complexes, supercoiled plasmid pUC19 DNA was used as a model to simulate the interactions. The supercoiled DNA showed a relatively faster migration speed in agarose gel electrophoresis due to its compact conformation. After addition of the complexes, the relaxed DNA appeared and a decrease in electrophoretic mobility was observed (Fig. S8†). When the two forms of DNA co-migrated, a coalescence point *r*_b(c)_ (the bound drug-to-nucleotide ratio) was reached, which corresponds to the full relaxation of the supercoiled DNA. The *r*_b(c)_ of OPT and PPT is 0.016 and 0.009, respectively (Table S2†). Cisplatin also produced a similar outcome, but the coalescence point is 0.076.^35^ The DNA unwinding ability of OPT and PPT is higher than that of cisplatin, and different from that of pyriplatin, which did not cause unwinding of the plasmid DNA.^27^ Since both OPT and pyriplatin formed monofunctional adducts (Fig. S9 and Table S3†), the difference in DNA unwinding ability is likely to arise from the –CH_2_Ph_3_P^+^ substituent and positive charges. Somehow, the DNA binding ability of MPT is very low.

mtDNA is more vulnerable than nDNA owing to the absence of histones and an insufficient DNA repair rate.^36^ Even so, the mtDNA damage caused by OPT was only about 2 times higher than that of cisplatin, which is inconsistent with the respective accumulation of Pt in mitochondria, indicating the mtDNA damage efficiency of OPT is lower than that of cisplatin. However, the *in vitro* and *in vivo* antitumor efficacy of OPT toward lung cancer cells and tissue was higher than that of cisplatin (Fig. 2), suggesting that some non-DNA pathways may be involved in the anticancer mechanism of OPT.

### Mitochondrial bioenergetics

**Fig. 4:**
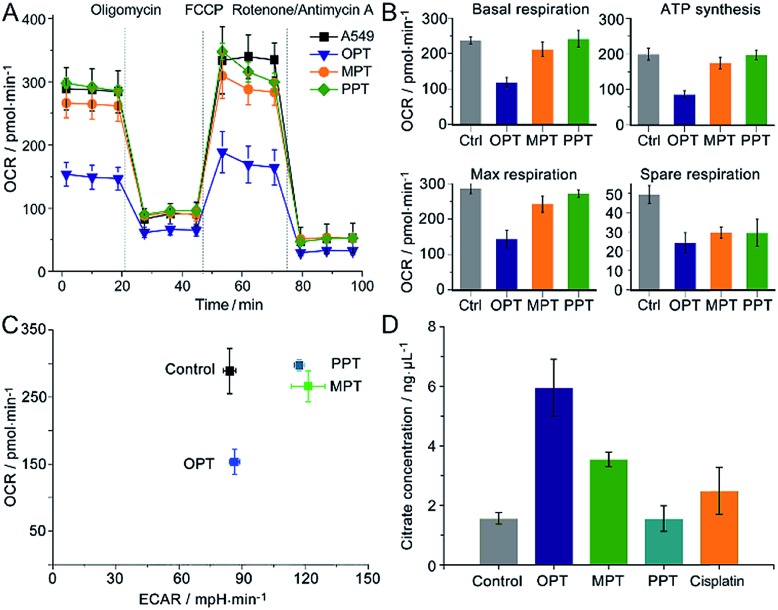
Mitochondrial bioenergetics in A549 cells. (A) OCR variations, (B) key parameters of mitochondrial respiration, (C) metabolic phenotypes and (D) intracellular citrate levels after treatment with OPT, MPT, PPT, and cisplatin (10 μM), respectively, for 24 h, presented as mean ± S.D. (*n* = 5).

The oxygen consumption rate (OCR) is an important indicator for mitochondrial functions.^37^ We therefore investigated the effect of OPT, MPT and PPT on the mitochondrial respiration of A549 cells. The cellular OCR was measured by an XF Cell Mito Stress Test, which could reflect the activity of mitochondrial OXPHOS. In the test, oligomycin was used to inhibit the ATP synthase and get the ATP synthetic ability of the cells; uncoupler FCCP was used to increase the respiration well above the basal level and get the maximal respiratory capacity; rotenone and antimycin A were used to inhibit the activities of complex I (NADH dehydrogenase) and complex III (cytochrome c reductase) respectively,^38^ so they can completely inhibit the electron transport chain. Multiple parameters of mitochondrial function were determined as reported previously.^39^ As shown in Fig. 4A and B, the level of basal respiration decreased evidently after A549 cells were incubated with OPT; the ATP synthesis and maximal respiration also decreased to much lower levels than those incubated with MPT and PPT. The effects on the spare respiration capacity are similar for these complexes.

In Fig. 4A, the basal respiration of the OPT-treated A549 cells is lower than that of the control, which might be caused by the dead cells or damaged mitochondria. To exclude the possible artefact, the effect of OPT and cisplatin on ATP synthesis was further investigated by the coupling assay using isolated mitochondria from muscles of wildtype C57BL/6 mice (8 weeks, female; Fig. S10†). The basal OCR in response to OPT and cisplatin was almost the same as that of the control. When FCCP was added, the maximal respiration of OPT-treated mitochondria was significantly decreased compared to that of cisplatin-treated mitochondria, confirming that the effect of OPT on the basal respiration is authentic.

The dynamic interplay between the two dominant energy-yielding pathways, mitochondrial respiration and glycolysis, was quantified by XF extracellular flux assay, which is a rapid and more comprehensive assessment of cellular bioenergetics.^40^ The basal OCR represents mitochondrial respiration, and the basal extracellular acidification rate (ECAR) reflects the glycolytic process. For A549 cells, the OCR decreased remarkably while the ECAR did not increase after the pretreatment with OPT as compared with MPT and PPT (Fig. 4C). The data showed that OPT can inhibit the mitochondrial OXPHOS, leading to weaker basal and maximal respirations and less ATP production, and prevent the glycolytic pathway of A549 cells simultaneously. In addition, the citrate level of OPT-treated cells increased more than that of cisplatin-treated cells (Fig. 4D). Citrate is a key intermediate of the tricarboxylic acid (TCA) cycle and an allosteric modulator of glycolysis.^41^ The mechanism of citrate elevation in the presence of a platinum complex is unknown at the moment. These results suggest that OPT has a great impact on the mitochondrial bioenergetics and the Warburg effect of cancer cells.

### MMP and ultrastructure

**Fig. 5:**
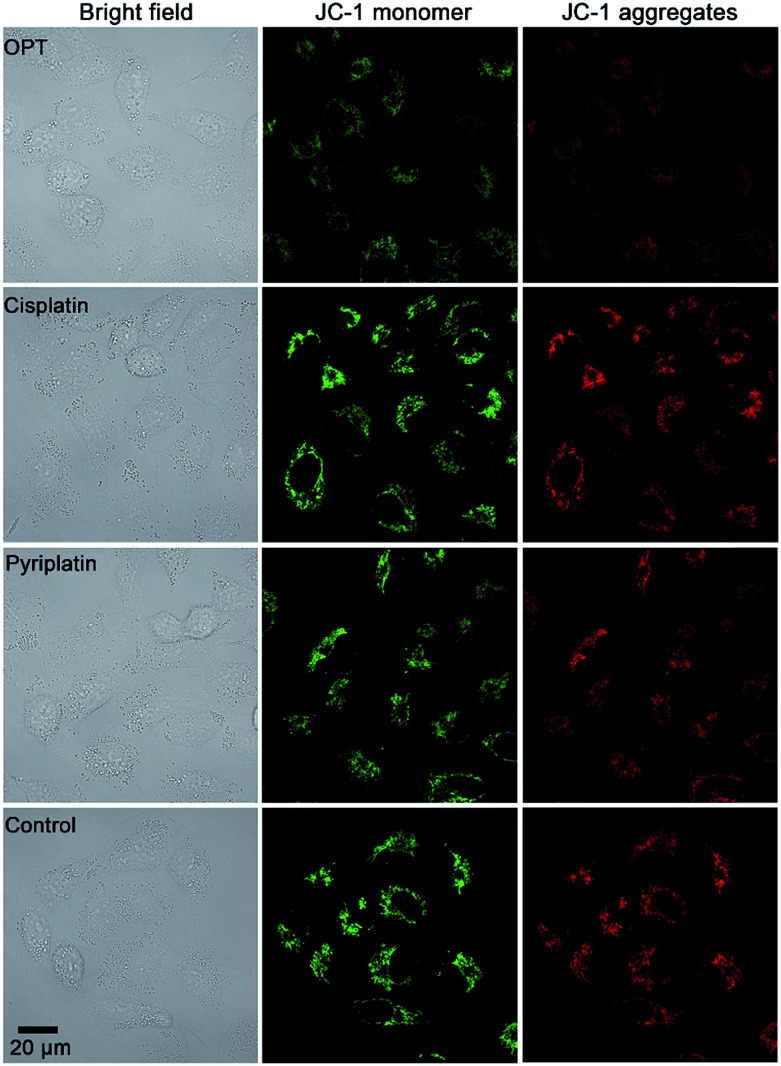
Representative images of A549 cells after incubation with OPT, cisplatin, and pyriplatin (10 μM) for 12 h, respectively, detected by fluorescence microscopy using a JC-1 probe.

MMP (Δ*Ψ*_m_) is an important physiologic parameter commonly used to monitor the cellular capacity for ATP generation *via* OXPHOS.^42^ The dissipation of MMP was detected by using a JC-1 fluorescent probe. Green (monomers) or red (aggregates) fluorescence of JC-1 were observed in the A549 cells, which reflects the effect of each complex on the Δ*Ψ*_m_ in mitochondria. As shown in Fig. 5, the fluorescence of JC-1 in A549 cells was significantly decreased after treatment with OPT for 12 h, while that in cells treated with cisplatin or pyriplatin barely changed as compared with the control. When the incubation time extended to 24 h, both OPT and cisplatin similarly depolarized the mitochondria (Fig. S11†), with the Δ*Ψ*_m_ being decreased 89% and 87%, respectively, relative to the negative control quantified by using Zone software. The results suggest that the depolarization of mitochondria induced by OPT occurred more rapidly than that induced by cisplatin due to the mitochondrion-targeting ability. The validity of JC-1 dye was confirmed by the protonophore CCCP, which depolarizes mitochondria by increasing their permeability to protons and losing the proton gradient (Fig. S11†).

**Fig. 6:**
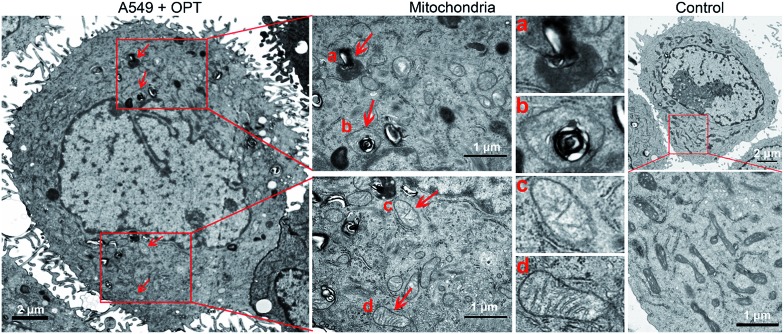
TEM images of the mitochondrial structure in A549 cells treated with OPT (10 μM) for 24 h and without treatment.

The morphological changes of mitochondria in A549 cells were investigated by transmission electron microscopy (TEM). As shown in Fig. 6, intact mitochondria have complete double membrane structures with an inner membrane and a smooth outer membrane. The polymorphic cristae within the mitochondria matrix is perpendicular to the axis of mitochondria. After exposure to OPT, the number of intact mitochondria decreased, the double membrane structure suffered certain damage, the cristae disappeared and excessive fragmentation appeared. Furthermore, numerous lamellar structures and double-membraned cytosolic autophagic vacuoles accumulated,^43^ like a bubble to degrade the organelle (Fig. 6a), and some autophagosomes with double membrane structure swelled the mitochondria (Fig. 6b).^44^ By contrast, cisplatin barely affected the morphology of mitochondria (Fig. S12†). In addition, after treatment with OPT, the number of mitochondria decreased, the cristae disappeared, and the double membrane structure of mitochondria was damaged. Since the number of mitochondria represents the level of cellular energy requirement, less healthy mitochondria in the OPT-treated cells imply that the energy metabolism is reduced. Furthermore, the complexes I–IV in the mitochondrial respiratory chain are distributed on the polymorphic cristae;^45^ the disappearance of cristae suggests that OPT could impair the mitochondrial respiration, which is consistent with the OCR data.

### Release of cytochrome c and apoptosis

**Fig. 7:**
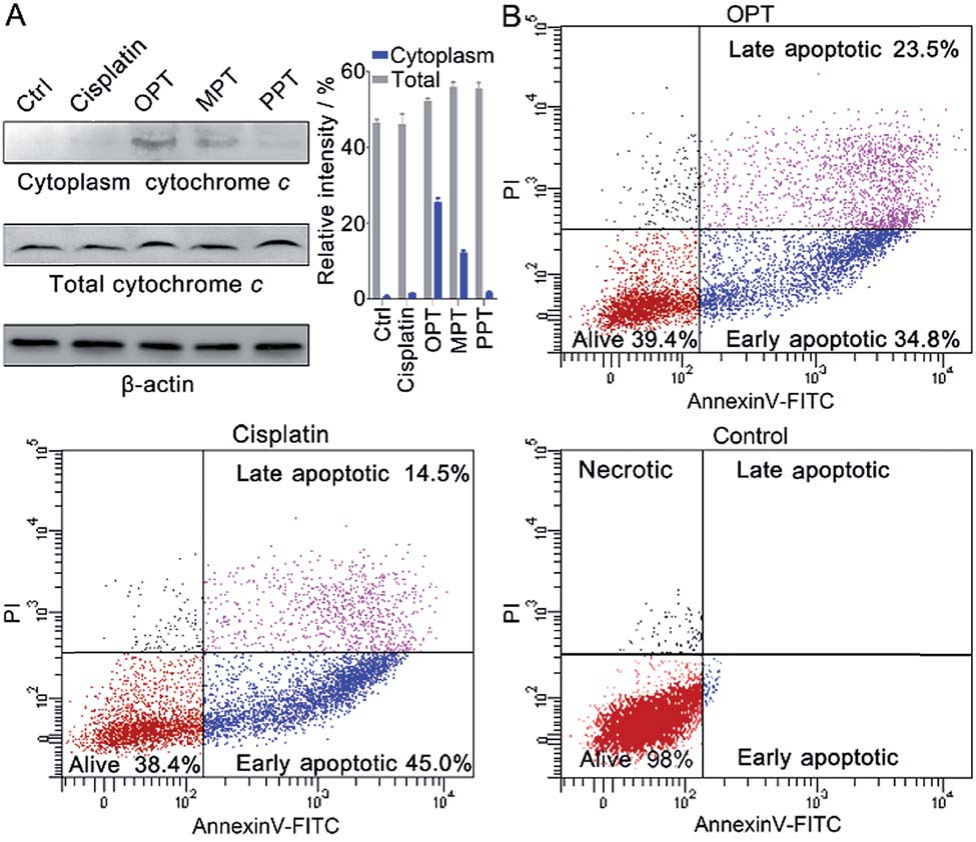
(A) Western blotting analysis of Cyto c released from the mitochondria of A549 cells after treatment with the complexes using β-actin (42 kDa) as a reference, and relative intensity of Cyto c analyzed by Image J (mean ± S.D., *n* = 3). (B) Flow cytometric analysis of A549 cells after incubation with OPT or cisplatin (10 μM) for 24 h and subsequent staining with Annexin V-FITC and PI.

In the presence of OPT, we observed that the Δ*Ψ*_m_ of A549 cells was dissipated, suggesting that the mitochondrial permeability transition pore (mPTP) was opened and the pro-apoptotic protein cytochrome c (Cyto c) was released, which is an early event of apoptosis.^46^,^47^ The presence of Cyto c in A549 cells was examined by Western blot analysis. As shown in Fig. 7A, OPT induced more Cyto c (14 kDa) release from mitochondria than MPT, PPT and cisplatin. The release of Cyto c from the mitochondria into the cytosol of A549 cells is a key event in the caspase activation.^48^ The executioner caspase may subsequently cleave some important intracellular substrates, leading to apoptosis.^49^

The apoptosis of A549 cells in the presence of OPT was detected by flow cytometric assay using Annexin V-FITC combined with propidium iodide (PI) staining.^50^ Apoptotic cells could be distinguished from intact cells according to Annexin V variations (Annexin V+ *vs.* Annexin V–). PI staining allows further distinction between early apoptotic (Annexin V+/PI–) and late apoptotic or necrotic (Annexin V+/PI+) cells. As shown in Fig. 7B, after treatment with OPT, 34.8% of A549 cells were in early apoptosis, and 23.5% of the cells were in late apoptosis, while 45.0% of the cells were in early apoptosis, and 14.5% of the cells were in late apoptosis after treatment with cisplatin. Although the changing trend of apoptosis induced by OPT and cisplatin is similar, OPT caused more late apoptosis than cisplatin. MPT and PPT mainly induced early apoptosis, but less efficient than OPT (Fig. S13†).

## Conclusions

**Fig. 8:**
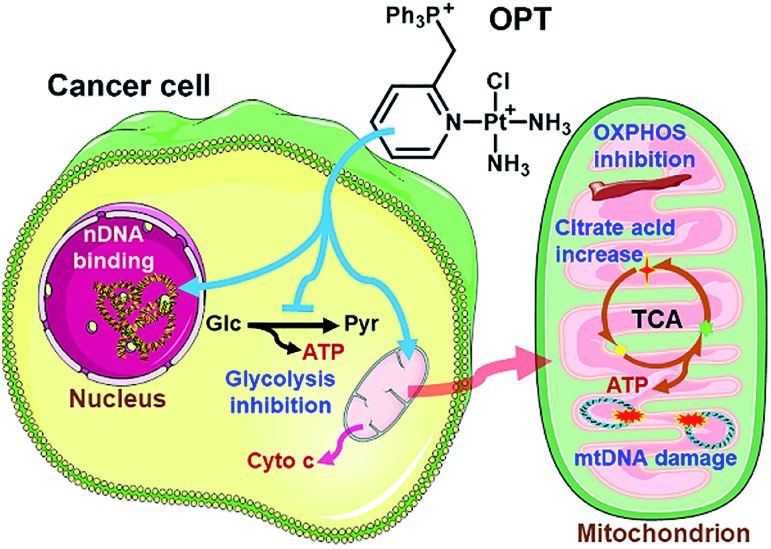
Proposed mechanism of action for OPT.

Three monofunctional mitochondrion-targeted Pt^II^ complexes were developed, and the biological reactivity was investigated. These complexes differ from each other according to the position of substituents, which significantly influences their cellular uptake and distribution. Among them, OPT exhibited remarkable anticancer potential toward A549 lung cancer cells and lung cancer xenograft in mice, while MPT and PPT were less potent than cisplatin; moreover, it was less toxic toward normal cells than cisplatin, implying a good biocompatibility. Unusual anticancer pathways involving nDNA/mtDNA interruption, mitochondrial bioenergetics, glycolysis inhibition and release of cytochrome c were revealed (Fig. 8), which provide new insights into the mechanism of action for platinum complexes. As mitochondria are related to energy metabolism and cell apoptotic machinery, mitochondrion-targeted Pt^II^ complexes may have potential to overcome the drug resistance of traditional platinum antitumor drugs.

## Conflicts of interest

There are no conflicts to declare.

## Supplementary Material

Supplementary information

## References

[cit1] Bray F., Ferlay J., Soerjomataram I., Siegel R. L., Torre L. A., Jemal A. (2018). Ca-Cancer J. Clin..

[cit2] Liu K.-J., Ding L.-Y., Wu H.-Y. (2015). Tumor Biol..

[cit3] Johnstone T. C., Suntharalingam K., Lippard S. J. (2016). Chem. Rev..

[cit4] Wang X. H., Wang X. Y., Jin S. X., Muhammad N., Guo Z. J. (2019). Chem. Rev..

[cit5] Brabec V., Hrabina O., Kasparkova J. (2017). Coord. Chem. Rev..

[cit6] Wang S.-S., Zimmermann M., Zhang H. Y., Lin T.-Y., Malfatti M., Haack K., Turteltaub K. W., Cimino G. D., de Vere White R., Pan C.-X., Henderson P. T. (2017). Int. J. Cancer.

[cit7] Langer C. J., Gadgeel S. M., Borghaei H., Papadimitrakopoulou V. A., Patnaik A., Powell S. F., Gentzler R. D., Martins R. G., Stevenson J. P., Jalal S. I., Panwalkar A., Yang J. C.-H., Gubens M., Sequist L. V., Awad M. M., Fiore J., Ge Y., Raftopoulos H., Gandhi L. (2016). Lancet Oncol..

[cit8] Macerelli M., Ganzinelli M., Gouedard C., Broggini M., Garassino M. C., Linardou H., Damia G., Wiesmüller L. (2016). Cancer Treat. Rev..

[cit9] Gatti L., Cassinelli G., Zaffaroni N., Lanzi C., Perego P. (2015). Drug Resist. Updates.

[cit10] Galluzzi L., Senovilla L., Vitale I., Michels J., Martins I., Kepp O., Castedo M., Kroemer G. (2012). Oncogene.

[cit11] Shen D.-W., Pouliot L. M., Hall M. D., Gottesman M. M. (2012). Pharmacol. Rev..

[cit12] Wallace D. C., Fan W., Procaccio V. (2010). Annu. Rev. Pathol.: Mech. Dis..

[cit13] Andreux P. A., Houtkooper R. H., Auwerx J. (2013). Nat. Rev. Drug Discovery.

[cit14] Lunt S. Y., Vander Heiden M. G. (2011). Annu. Rev. Cell Dev. Biol..

[cit15] Hamanaka R. B., Chandel N. S. (2011). Science.

[cit16] Upadhyay M., Samal J., Kandpal M., Singh O. V., Vivekanandan P. (2013). Pharmacol. Ther..

[cit17] Liberti M. V., Locasale J. W. (2016). Trends Biochem. Sci..

[cit18] Yang Y. H., Karakhanova S., Hartwig W., D'Haese J. G., Philippov P. P., Werner J., Bazhin A. V. (2016). J. Cell. Physiol..

[cit19] Bhat T. A., Kumar S., Chaudhary A. K., Yadav N., Chandra D. (2015). Drug Discovery Today.

[cit20] Guaragnella N., Giannattasio S., Moro L. (2014). Biochem. Pharmacol..

[cit21] Weinberg S. E., Chandel N. S. (2015). Nat. Chem. Biol..

[cit22] Wisnovsky S. P., Wilson J. J., Radford R. J., Pereira M. P., Chan M. R., Laposa R. R., Lippard S. J., Kelley S. O. (2013). Chem. Biol..

[cit23] Wang F., Ogasawara M. A., Huang P. (2010). Mol. Aspects Med..

[cit24] Smith R. A. J., Hartley R. C., Murphy M. P. (2011). Antioxid. Redox Signaling.

[cit25] Marrache S., Pathak R. K., Dhar S. (2014). Proc. Natl. Acad. Sci. U. S. A..

[cit26] Jin S. X., Hao Y. G., Zhu Z. Z., Muhammad N., Zhang Z. Q., Wang K., Guo Y., Guo Z. J., Wang X. Y. (2018). Inorg. Chem..

[cit27] Lovejoy K. S., Todd R. C., Zhang S. Z., McCormick M. S., Alejandro D'Aquino J., Reardon J. T., Sancar A., Giacomini K. M., Lippard S. J. (2008). Proc. Natl. Acad. Sci. U. S. A..

[cit28] Park G. Y., Wilson J. J., Song Y., Lippard S. J. (2012). Proc. Natl. Acad. Sci. U. S. A..

[cit29] Dresser M. J., Leabman M. K., Giacomini K. M. (2001). J. Pharm. Sci..

[cit30] Sancho-Martinez S. M., Prieto-Garcia L., Prieto M., Lopez-Novoa J. M., Lopez-Hernandez F. J. (2012). Pharmacol. Ther..

[cit31] Hofmann J. N., Hosgood III H. D., Liu C.-S., Chow W.-H., Shuch B., Cheng W.-L., Lin T.-T., Moore L. E., Lan Q., Rothman N., Purdue M. P. (2014). Carcinogenesis.

[cit32] Shaughnessy D. T., McAllister K., Worth L., Haugen A. C., Meyer J. N., Domann F. E., Van Houten B., Mostoslavsky R., Bultman S. J., Baccarelli A. A., Begley T. J., Sobol R. W., Hirschey M. D., Ideker T., Santos J. H., Copeland W. C., Tice R. R., Balshaw D. M., Tyson F. L. (2014). Environ. Health Perspect..

[cit33] Galluzzi L., Kepp O., Kroemer G. (2016). Microb. Cell.

[cit34] Rothfuss O., Gasser T., Patenge N. (2010). Nucleic Acids Res..

[cit35] Keck M. V., Lippard S. J. (1992). J. Am. Chem. Soc..

[cit36] Wallace D. C. (2012). Nat. Rev. Cancer.

[cit37] Zhdanov A. V., Waters A. H. C., Golubeva A. V., Dmitriev R. I., Papkovsky D. B. (2014). Biochim. Biophys. Acta, Bioenerg..

[cit38] Abe Y., Sakairi T., Kajiyama H., Shrivastav S., Beeson C., Kopp J. B. (2010). Am. J. Physiol.: Cell Physiol..

[cit39] Zhang J., Nuebel E., Wisidagama D. R. R., Setoguchi K., Hong J. S., Van Horn C. M., Imam S. S., Vergnes L., Malone C. S., Koehler C. M., Teitell M. A. (2012). Nat. Protoc..

[cit40] Rogers G. W., Brand M. D., Petrosyan S., Ashok D., Elorza A. A., Ferrick D. A., Murphy A. N. (2011). PLoS One.

[cit41] Costello L. C., Franklin R. B. (2013). J. Regener. Med. Tissue Eng..

[cit42] Vander Heiden M. G., Cantley L. C., Thompson C. B. (2009). Science.

[cit43] Guo W.-J., Zhang Y.-M., Zhang L., Huang B., Tao F.-F., Chen W., Guo Z.-J., Xu Q., Sun Y. (2013). Autophagy.

[cit44] Gomes L. C., Di Benedetto G., Scorrano L. (2011). Nat. Cell Biol..

[cit45] Cogliati S., Frezza C., Soriano M. E., Varanita T., Quintana-Cabrera R., Corrado M., Cipolat S., Costa V., Casarin A., Gomes L. C., Perales-Clemente E., Salviati L., Fernandez-Silva P., Enriquez J. A., Scorrano L. (2013). Cell.

[cit46] Sarraf S. A., Raman M., Guarani-Pereira V., Sowa M. E., Huttlin E. L., Gygi S. P., Harper J. W. (2013). Nature.

[cit47] McGill M. R., Sharpe M. R., Williams C. D., Taha M., Curry S. C., Jaeschke H. (2012). J. Clin. Invest..

[cit48] Li H., Wang P., Sun Q. H., Ding W.-X., Yin X.-M., Sobol R. W., Stolz D. B., Yu J., Zhang L. (2011). Cancer Res..

[cit49] Westermann B. (2010). Nat. Rev. Mol. Cell Biol..

[cit50] Muhammad N., Wang X. Y., Wang K., Zhu C. C., Zhu Z. Z., Jiao Y., Guo Z. J. (2016). Dalton Trans..

