# Supplementary material for: Mitochondrion-targeted platinum complexes suppressing lung cancer through multiple pathways involving energy metabolism
Source: Chem Sci. 2019 Jan 22;10(10):3089–95. doi: 10.1039/c8sc04871a (PMC6428137; doi:10.1039/c8sc04871a)
Supplement: Supplementary file 1 [file SC-010-C8SC04871A-s001.pdf]

Electronic Supplementary Material (ESI) for Chemical Science.
This journal is © The Royal Society of Chemistry 2019

Electronic Supplementary Information (ESI)

Mitochondrion-targeted platinum complexes suppressing lung cancer through multiple pathways involving energy metabolism

Zhenzhu Zhu, Zenghui Wang, Changli Zhang, Yanjun Wang, Hongmei Zhang, Zhenji Gan, Zijian Guo* and Xiaoyong Wang*

1. Supplementary figures and tables

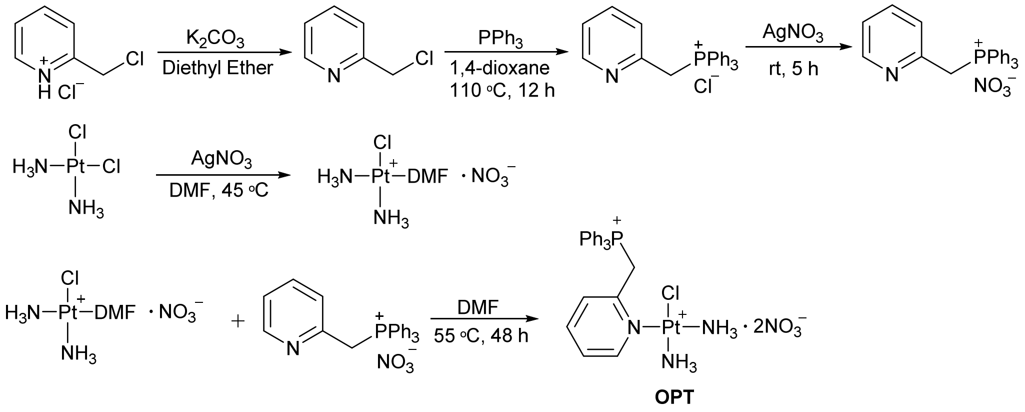

Scheme S1. Synthetic route to OPT.

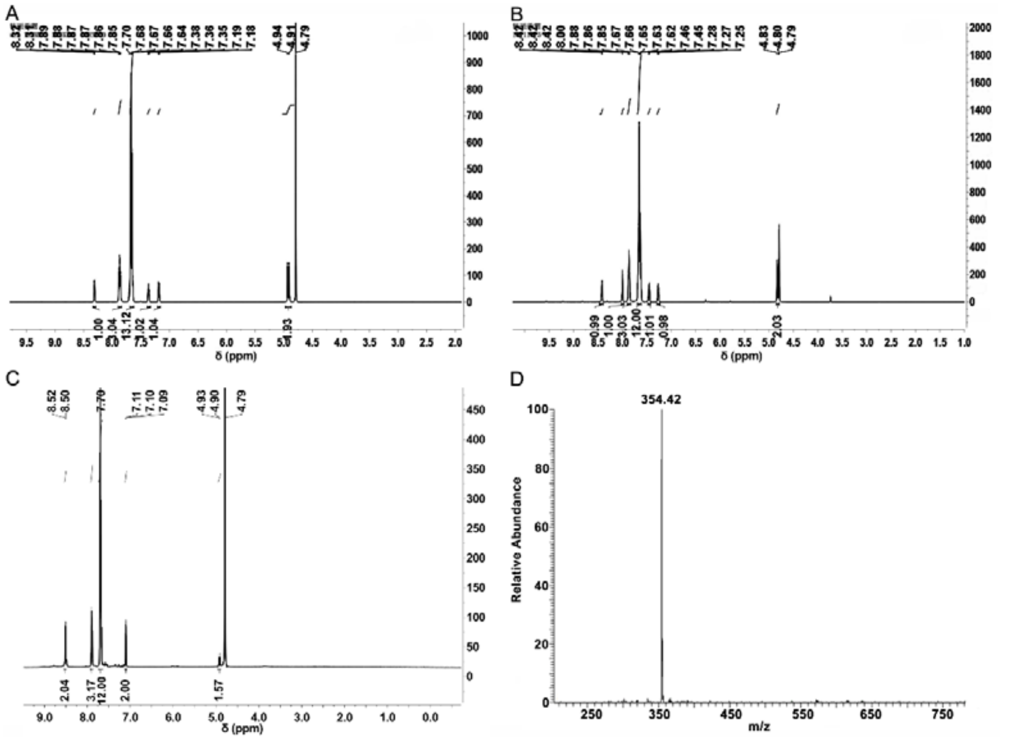

Fig. S1 ^{1}H-NMR spectra (500 MHz, D_2O) of *o*-PPh_3CH_2PyCl (A), *m*-PPh_3CH_2PyCl (B), *p*-PPh_3CH_2PyCl (600 MHz, D_2O) (C), and ESI-MS spectrum of *o*-PPh_3CH_2PyCl (D).

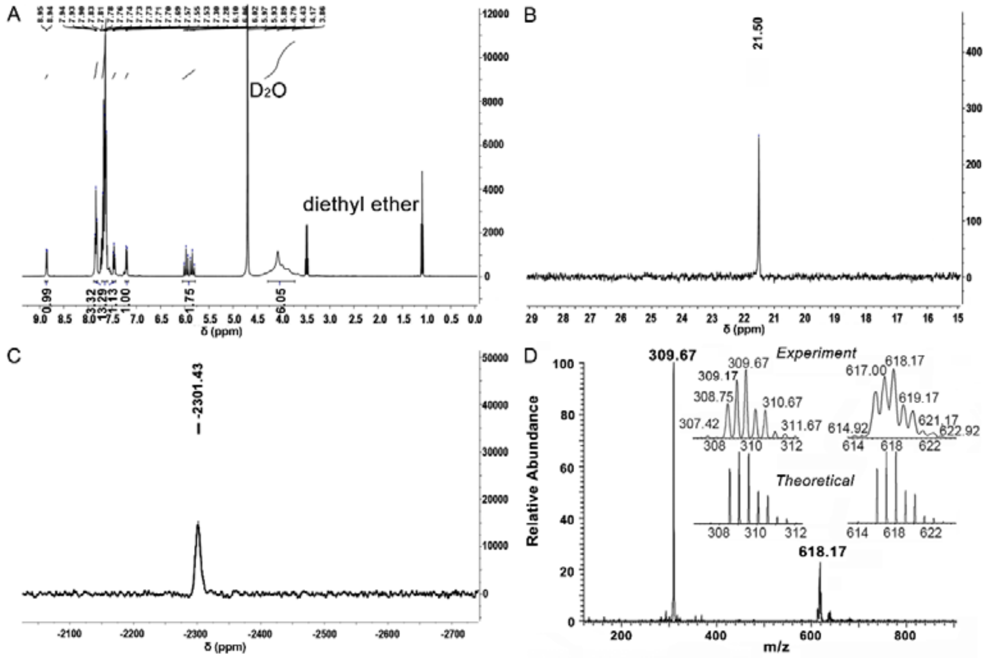

Fig. S2 ^{1}H-NMR (400 MHz, D_2O) (A), ^{31}P-NMR (202 MHz, D_2O) (B), ^{195}Pt-NMR (107 MHz, DMSO-d^6) (C) and ESI-MS spectra of OPT (D).

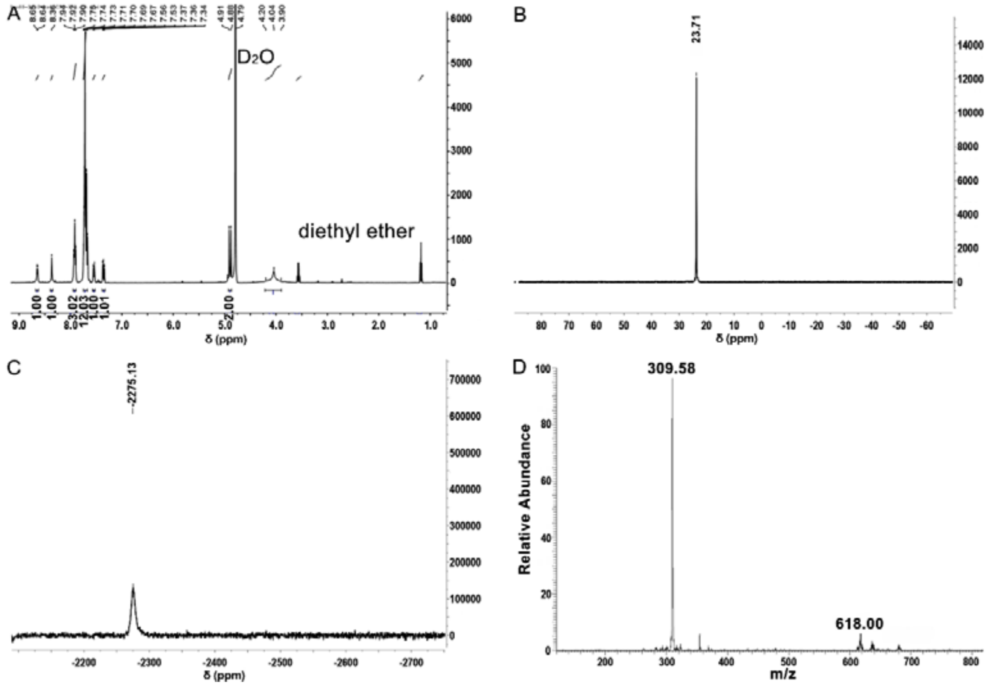

Fig. S3 ^{1}H-NMR (400 MHz, D_2O) (A), ^{31}P-NMR (202 MHz, DMSO-d^6) (B), ^{195}Pt-NMR (107 MHz, DMSO-d^6) (C) and ESI-MS spectra of MPT (D).

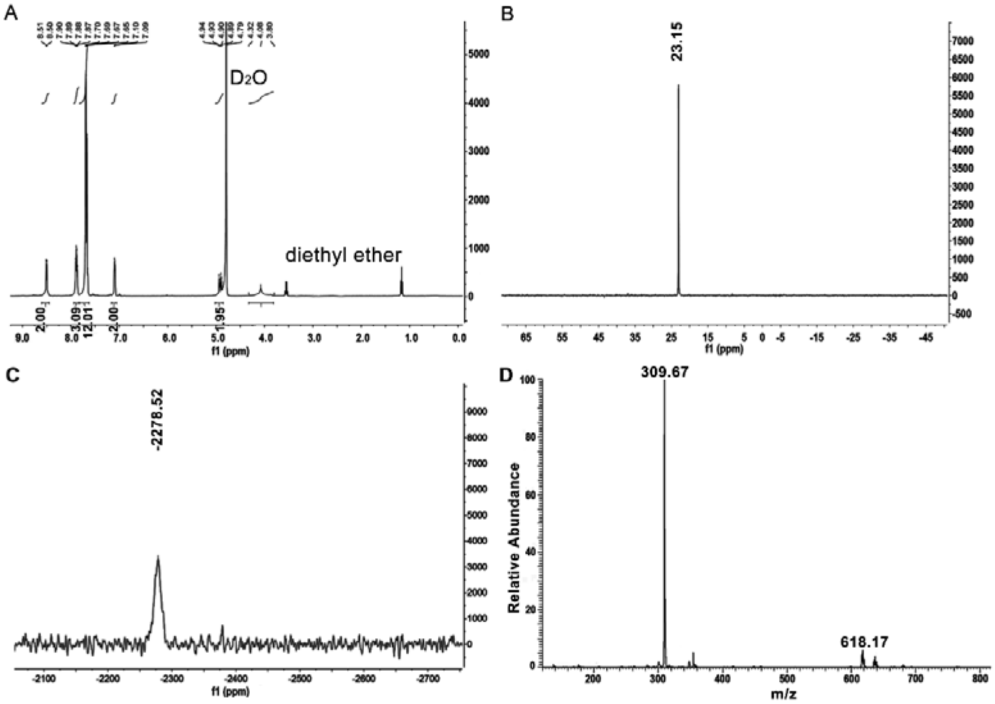

Fig. S4 ^{1}H-NMR (400 MHz, D_2O) (A), ^{31}P-NMR (202 MHz, DMSO-d^{6}) (B), ^{195}Pt-NMR (107 MHz, DMSO-d^{6}) (C) and ESI-MS spectra of PPT (D).

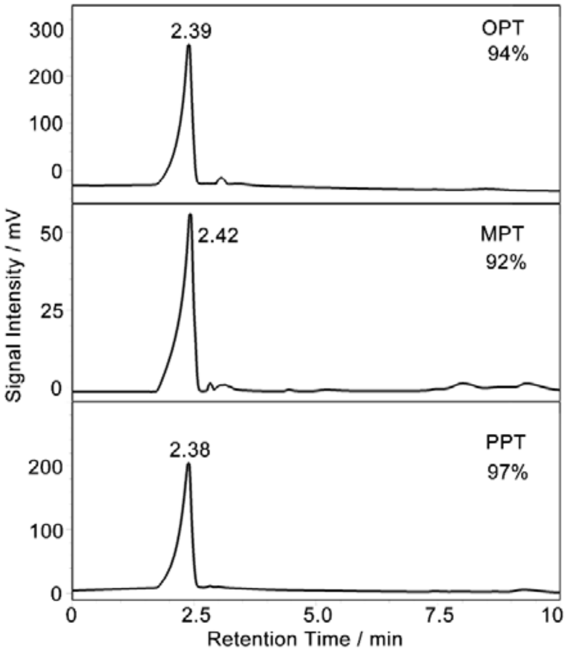

Fig. S5 HPLC for OPT, MPT and PPT recorded at 230 nm on a Beckman Coulter HPLC instrument equipped with a C18 reverse phase column (eluent: CH_3OH/H_2O, 7/3).

Table S1. IC_{50} values of the complexes against different cell lines after incubation for 48 h.

	A549	SMMC	HeLa	HL-7702
OPT	8.7 ± 1.6	12.2 ± 0.9	20.2 ± 1.7	64.5 ± 3.2
MPT	47.8 ± 4.5	85.8 ± 3.3	23.6 ± 1.4	> 200
PPT	23.5 ± 2.7	> 200	34.6 ± 3.4	53.7 ± 2.9
Cisplatin	12.6 ± 1.1	7.6 ± 0.6	18.1 ± 0.7	14.8 ± 2.3
Pyriplatin	125.5 ± 5.8	/	/	/

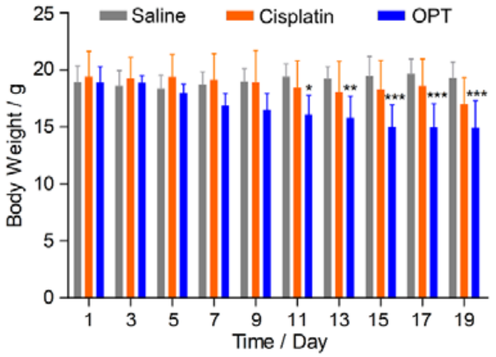

Fig. S6 Time-course for body weight of mice (n = 5) treated by OPT, cisplatin, and saline respectively for 19 days, $*p < 0.1$, $**p < 0.01$, $***p < 0.001$.

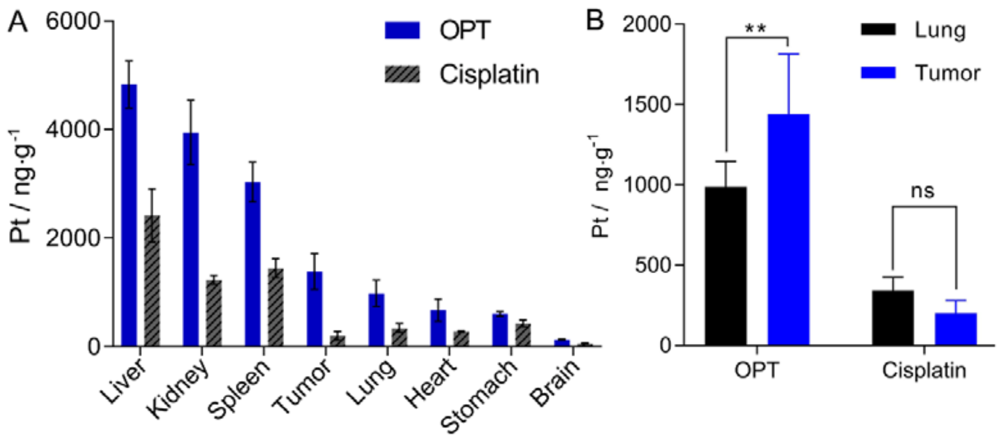

Fig. S7 Biodistribution of OPT and cisplatin in terms of Pt in major organs of mice after 19 days treatment determined by ICP-MS (A), and the distribution of OPT and cisplatin in terms of Pt in normal lung and tumor tissue (B). Error bars indicate standard deviations, n = 5; $**p < 0.01$, ns = not significant.

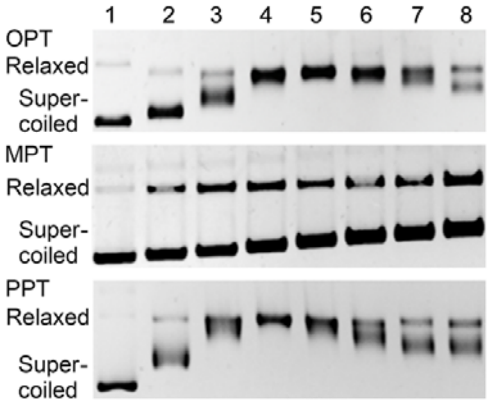

Fig. S8 Agarose gel electrophoresis patterns of supercoiled pUC19 plasmid DNA (200 ng) incubated with different Pt complexes at 37 °C for 24 h. OPT: Lane 1, control, Lanes 2–8, r_b = 0.004, 0.008, 0.010, 0.016, 0.018, 0.025, 0.035, respectively; MPT: Lane 1, control, Lanes 2–8, r_b = 0.006, 0.008, 0.010, 0.010, 0.011, 0.014, 0.017, respectively; PPT: Lane 1, control, Lanes 2–8, r_b = 0.003, 0.007, 0.009, 0.011, 0.015, 0.018, 0.020, respectively.

Table S2. Formal drug-to-nucleotide ratios (r_f) and the bound drug-to-nucleotide ratios (r_b) of the complexes.

No.	OPT		MPT		PPT	
	r_f	r_b	r_f	r_b	r_f	r_b
1	0	0	0	0	0	0
2	0.32	0.004 ± 0.0001	0.32	0.006 ± 0.0002	0.16	0.003 ± 0.0002
3	0.64	0.008 ± 0.0003	0.64	0.008 ± 0.0002	0.32	0.007 ± 0.0004
4	0.96	0.010 ± 0.0008	0.96	0.010 ± 0.0003	0.48	0.009 ± 0.0011
5	1.28	0.016 ± 0.0018	1.28	0.010 ± 0.0001	0.64	0.011 ± 0.0007
6	1.60	0.018 ± 0.0012	1.60	0.011 ± 0.0010	0.80	0.015 ± 0.0022
7	1.92	0.025 ± 0.0019	1.92	0.014 ± 0.0024	0.96	0.018 ± 0.0013
8	2.24	0.035± 0.0011	2.24	0.017 ± 0.0003	1.12	0.020 ± 0.0009

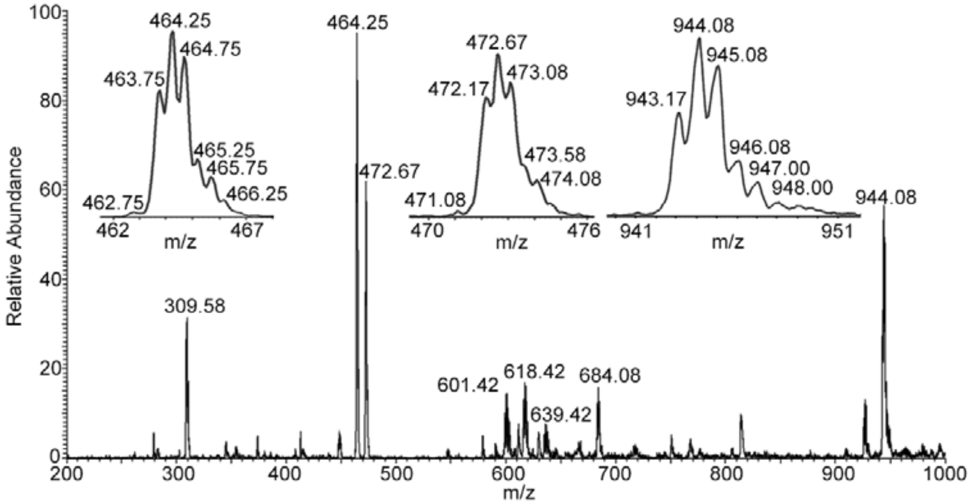

Fig. S9 ESI-MS spectrum (positive mode) of the reaction between OPT and 5′-GMP (1: 2) recorded in water at 37 °C for 24 h.

Table S3. Assignments of ESI-MS spectrum of the reaction between OPT and 5′-GMP (1: 2) recorded in water at 37 °C for 24 h.

Assignments	Chemical Formula	Cal. m/z	Exp. m/z
[Pt(*o*-PPh$_3$CH$_2$Py)(NH$_3$)$_2$ (GMP)-OH]$^{2+}$	$C_{34}H_{40}N_8O_7P_2Pt$	464.61	462.75–466.25
[Pt(*o*-PPh$_3$CH$_2$Py) (NH$_3$)$_2$ (GMP)+H]$^{2+}$	$C_{34}H_{41}N_8O_8P_2Pt$	473.11	471.08–475.08
[Pt(*o*-PPh$_3$CH$_2$Py) (NH$_3$)$_2$ (GMP)]$^{+}$	$C_{34}H_{40}N_8O_8P_2Pt$	945.21	943.17–948.00

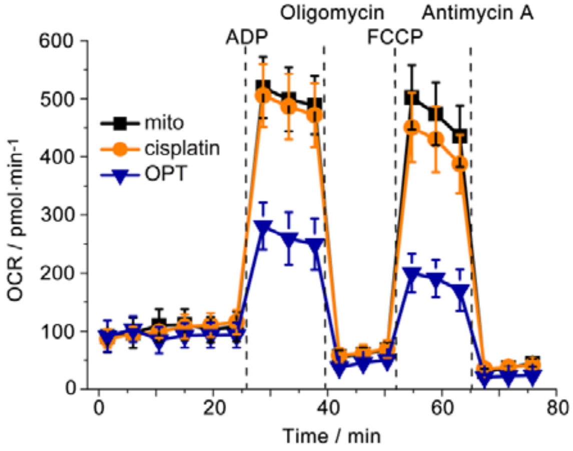

Fig. S10 OCR variations of isolated mitochondria from muscles of wildtype C57BL/6 mice (8 weeks, female) measured after mitochondria (4 μg) were incubated with OPT and cisplatin (10 μM) respectively for 30 min. Data are presented as mean ± S.D., n = 5.

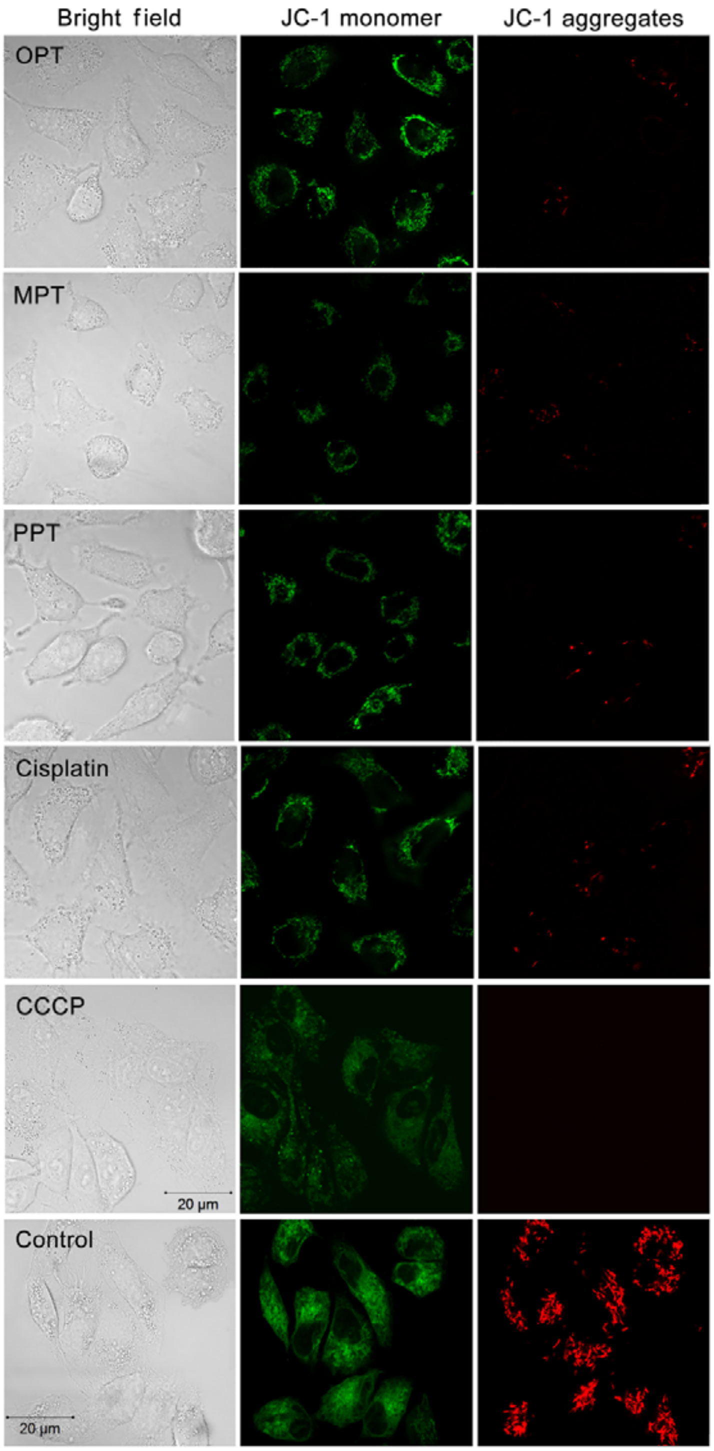

Fig. S11 Representative images of A549 cells after incubation with OPT, MPT, PPT, and cisplatin (10 μM) for 24 h, or CCCP (50 nM) for 10 min, respectively, detected by fluorescence microscope using JC-1 probe.

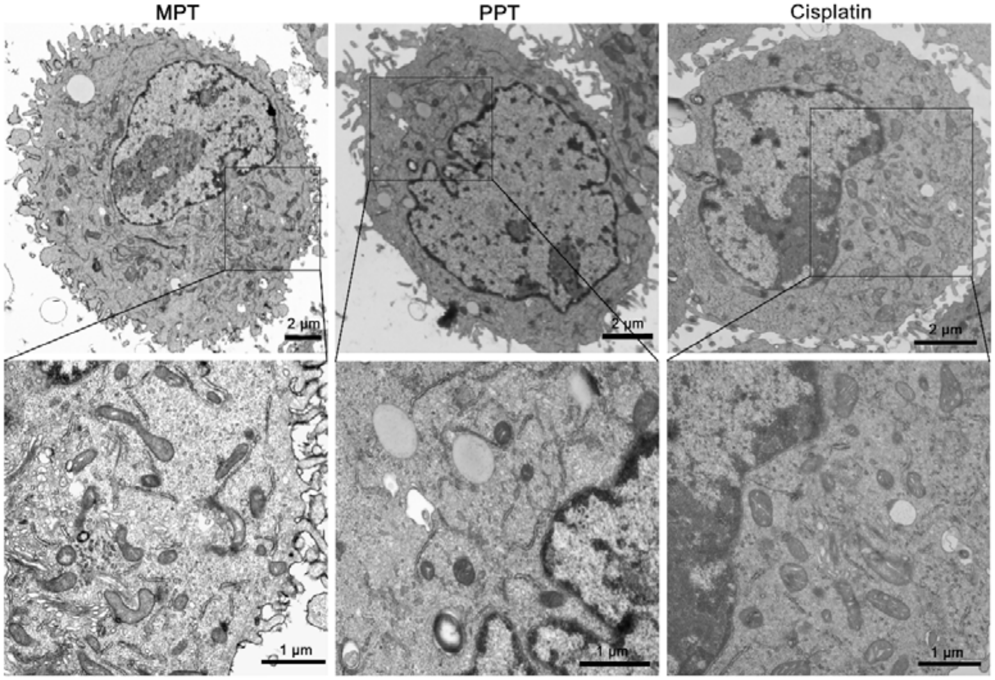

Fig. S12 TEM images of mitochondria structure of A549 cells treated with MPT, PPT, and cisplatin, respectively.

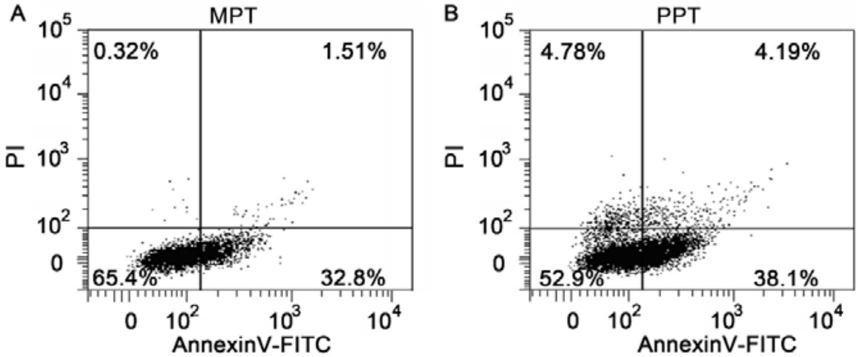

Fig. S13 Flow cytometric analysis of A549 cells incubated with 10 μM of MPT (A) or PPT (B) for 24 h, and subsequent staining with Annexin V-FITC and PI.

2. Experimental

2.1 Chemical reagents

Potassium carbonate, 2-(chloromethyl) pyridine hydrochloride, 3-(chloromethyl) pyridine hydrochloride, 4-(chloromethyl) pyridine hydrochloride, 1,4-dioxane, triphenylphosphonium chloride, silver nitrate, anhydrous Na_2SO_4, and 3-(4,5-dimethylthiazol-2-yl)-2,5-diphenyltetrazoliumbromide (MTT) were purchased from Sigma. Nitric acid, hydrogen peroxide (30%), and hydrochloric acid were

purchased from J&K. Cisplatin was purchased from Shandong Boyuan Pharmaceutical Co., Ltd.. PUC19 plasmid DNA was purchased from Thermo Fisher Scientific (USA). Glucose, pyruvate, oligomycin, carbonyl cyanide-p-trifluoromethoxyphenylhydrazone (FCCP), rotenone, antimycin A, succinate, and adenosine diphosphate (ADP) were purchased from Sigma. Glutaric dialdehyde (2.5%), uranyl acetate, lead citrate were obtained from Nanjing Medical University. Agarose, 30% acrylamide and sodium dodecyl sulfate (SDS) were purchased from Sunshinbio Ltd.. β-Actin mouse monoclonal-antibody was purchased from Sigma, USA. Cytochrome *c* rabbit monoclonal-antibody was purchased from Cell Signaling Technology, MA, USA. The secondary antibody, goat anti-mouse IgG H&L (HRP) and goat anti-rabbit IgG H&L (HRP) were purchased from Abcam, Cambridge, MA, USA. Immobilon western chemiluminescent HRP substrate was purchased from Millipore, Billerica, USA.

Mitochondria isolation kit was purchased from KeyGEN Co., Nanjing. TIANamp Genomic DNA kit was purchased from Tiangen (Beijing) Biotech. SsoFast EvaGreen Supermix (Bio-Rad) was used according to the manufacturer's protocol. XF24 cell mito-stress test kit and XF assay medium were purchased from Seahorse Bioscience, Billerica, MA. Pierce™ Coomassie (Bradford) protein assay kit was purchased from Thermo Fisher Scientific (USA). MuCyte™ mitochondrial isolatation kit was purchased from MuCyte BioTech Co., Nanjing. Citrate assay kit was purchased from Sigma. 5,5′,6,6′-Tetrachloro-1,1′,3,3′-tetraethylbenzimidazolylcarbo-cyanine iodide (JC-1) mitochondrial membrane potential assay kit containing carbonyl cyanide *m*-chlorophenyl hydrazone (CCCP) was purchased from KeyGEN Co., Nanjing. FITC Annexin V apoptosis detection kit was purchased from BD Pharmingen™ (Becton Dickinson Company, USA).

2.2 Cell lines and animals

The human non-small-cell lung cancer A549, human cervical cancer HeLa, human liver cancer SMMC and human normal liver HL-7702 cell lines were purchased from American Type Culture Collection (ATCC). The cells were cultured in RPMI-1640 medium supplemented with 10% heat-inactivated fetal bovine serum in 5% CO_2 atmosphere at 37 °C. Wildtype C57BL/6 mice and STOCK-Foxn1^{nu}/Nju nude mice were purchased from the Model Animal Research Center of Nanjing University.

2.3 Instruments

^{1}H-, ^{31}P- and ^{195}Pt-NMR spectra were recorded at 298 K on Bruker DRX 400 MHz, 500 MHz or 600 MHz NMR spectrometer, using tetramethylsilane as an external reference (δ = 0 ppm). Electrospray ionization mass spectrometry (ESI-MS) spectra were recorded using an LCQ fleet ESI-MS spectrometer (Thermo Scientific) in the positive mode, and the isotopic distribution patterns of the observed species were simulated using the Isopro 3.0 program. HPLC was carried out on a Beckman Coulter HPLC instrument equipped with a C18 reverse phase column. The data of MTT assay were determined using a Tecan Sunrise ELISA Reader at 570 nm. The content of Pt was determined on an inductively coupled plasma mass spectrometer (ICP-MS) using a standard Plasma-Quad II instrument (VG Elemental, Thermo OptekCorp.). The images of agarose gel electrophoresis were obtained by using a Bio-Rad Gel-Doc XR imaging system. UV-Vis absorption spectra were recorded on a Perkin-Elmer Lambda-35 UV-Vis spectrophotometer using quartz cuvettes (1.0 cm). Real-time PCR assay was performed in a Bio-Rad CFX96TM Real-Time PCR Detection System (Bio-Rad Laboratories, Hercules, CA). OCR was measured on the XF24 Extracellular Flux Analyzer (Seahorse Bioscience, Billerica, MA). Fluorescence confocal imaging was carried out on a laser scanning confocal imaging system (Olympus TH4-200) consisting of ZEISS Laser Scanning Microscope (LSM 710) and a 20 mW-output 488 nm argon ion laser. Ultrathin sections were cut with glass knives using a LKB ultra-microtome with 80 nm section thickness, and cell samples were collected on copper grids. TEM image was obtained using a JEOL JEM-2100 transmission electron microscope. Western blotting was carried on the Bio-Rad mini-PROTEAN tetra system and Bio-Rad Powerpack Universal. Images were captured using a Chemiscope 3400 mini (Clinx science instrument co. Ltd). Flow cytometry was measured by BD LSRFortessa^{TM} (Amersham Biosciences Corp.).

2.4 Synthesis of complexes

Ligand *o*-PPh_3CH_2PyCl was prepared as follows.[1] K_2CO_3 (3.37 g, 24.40 mmol) was added into 2-(chloromethyl)pyridine hydrochloride (8.00 g, 48.80 mmol) solution to adjust the pH to 7. The reaction mixture was extracted with diethyl ether, dried with anhydrous Na_2SO_4 and concentrated. The residue (5.60 g, 43.90 mmol) was dissolved in 1,4-dioxane (25 mL), and triphenylphosphine (11.54 g, 44 mmol) was

added. The mixture was heated at 110 °C, refluxed for 12 h, and filtered to obtain the solid product, which was washed with diethyl ether and dried under vacuum to get the deep yellow powder. Yield: 16.90 g, 89%. ^{1}H-NMR of *o*-PPh_3CH_2PyCl (500 MHz, D_2O): δ (ppm) 4.91–4.94 (d, 2H, -CH_2-), 7.18–7.19 (d, 1H, py-H), 7.35–7.38 (t, 1H, py-H), 7.64–7.70 (m, 13H, Ph-H), 7.85–7.89 (m, 2H, Ph-H, 1H, py-H), 8.31–8.32 (d, 1H, py-H). ESI-MS (positive mode, m/z) found (calcd): 354.42 (354.14), [*o*-$PPh_3CH_2Py]^{+}$. Ligands *m*-PPh_3CH_2PyCl and *p*-PPh_3CH_2PyCl were synthesized similarly except 3-(chloromethyl)pyridine hydrochloride and 4-(chloromethyl)-pyridine hydrochloride were used, respectively. ^{1}H-NMR of *m*-PPh_3CH_2PyCl (500 MHz, D_2O): δ (ppm) 4.80–4.83 (d, 2H, -CH_2-), 7.25–7.28 (t, 1H, py-H), 7.45–7.46 (d, 1H, py-H), 7.62–7.67 (m, 12H, Ph-H), 7.85–7.88 (t, 3H, Ph-H), 8.00 (s, 1H, py-H), 8.42 (s, 1H, py-H). ESI-MS (positive mode, m/z) found (calcd): 354.42 (354.14), [*m*-$PPh_3CH_2Py]^{+}$. ^{1}H-NMR of *p*-PPh_3CH_2PyCl (600 MHz, D_2O): δ (ppm) 4.90–4.93 (q, 2H, -CH_2-), 7.09–7.11 (m, 2H, py-H), 7.67–7.71 (m, 12 H, Ph-H), 7.88–7.91 (m, 3H, py-H), 8.50–8.52 (d, 2H, py-H). ESI-MS (positive mode, m/z) found (calcd): 354.42 (354.14), [*p*-$PPh_3CH_2Py]^{+}$.

Complex OPT was synthesized as follows.[2] *o*-PPh_3CH_2PyCl (150 mg, 0.39 mmol) was dissolved in anhydrous DMF (5 mL) and reacted with $AgNO_3$ (66 mg, 0.39 mmol) under stirring for 5 h at room temperature. The yellow supernatant containing *o*-$PPh_3CH_2PyNO_3$ was obtained after centrifugation. Cisplatin (150 mg, 0.5 mmol) and $AgNO_3$ (80 mg, 0.47 mmol) were stirred in anhydrous DMF (3 mL) overnight in the dark at 45 °C. A pale yellow solution of *cis*-[Pt(NH_3)$_2$Cl(DMF)](NO_3) was obtained after centrifugation. The above *o*-$PPh_3CH_2PyNO_3$ solution was dropped into [*cis*-Pt(NH_3)$_2$Cl(DMF)](NO_3) solution and stirred for 48 h in the dark at 55 °C. The resulting golden solution was filtered and evaporated. The oily substance was rinsed by CH_2Cl_2, extracted with hot methanol (100 mL), and the extract was concentrated to 5 mL. A light yellow precipitate was obtained after addition of diethyl ether, which was washed with CH_2Cl_2 and diethyl ether, dried in vacuum to obtain the final product OPT. Yield: 136 mg, 47%. ^{1}H-NMR of OPT (400 MHz, D_2O): δ (ppm) 3.86–4.43 (broad, 6H, -NH_3), 5.89–6.10 (m, 2H, -CH_2-), 7.28–7.30 (d, 1H, py-H), 7.53–7.57 (m, 1H, py-H), 7.69–7.83 (m, 13 H, Ph-H), 7.90–7.94 (m, 2H, Ph-H, 1H, py-H), 8.94–8.95 (d, 1H, py-H). ^{31}P-NMR (202 MHz, D_2O): δ (ppm) 21.50. ^{195}Pt-NMR (107 MHz, DMSO-d^{6}): δ (ppm) –2301.43. ESI-MS (positive mode, m/z)

found (calcd): 307.42–312.17 (309.06), [Pt(*o*-PPh_3CH_2Py)$(NH_3)_2Cl$]$^{2+}$; 614.92–622.92 (619.01), [Pt(*o*-PPh_3CH_2Py)$(NH_3)_2Cl$−H]$^{+}$. Purity (HPLC): 94% (retention time: 2.39 min, eluent: CH_3OH/H_2O = 7/3). MPT and PPT were synthesized by similar procedures except using *m*-PPh_3CH_2PyCl and *p*-PPh_3CH_2PyCl as the ligands, respectively. ^{1}H-NMR of MPT (400 MHz, D_2O): δ (ppm) 3.90–4.20 (broad, 6H, -NH_3), 4.88–4.91 (d, 2H, -CH_2-), 7.34–7.37 (t, 1H, py-H), 7.53–7.56 (d, 1H, py-H), 7.67–7.75 (m, 12H, Ph-H), 7.90–7.94 (m, 3H, Ph-H), 8.36 (s, 1H, py-H), 8.64–8.65 (d, 1H, py-H). ^{31}P-NMR (202 MHz, DMSO-d^6): δ (ppm) 23.71. ^{195}Pt-NMR (107 MHz, DMSO-d^6): δ (ppm) –2275.13. ESI-MS (positive mode, m/z) found (calcd): 307.17–312.17 (309.06), [Pt(*m*-PPh_3CH_2Py)$(NH_3)_2Cl$]$^{2+}$; 614.75–622.67 (619.01), [Pt(*m*-PPh_3CH_2Py)$(NH_3)_2Cl$−H]$^{+}$. Purity (HPLC): 92% (retention time: 2.42 min, eluent: CH_3OH/H_2O = 7/3). ^{1}H-NMR of PPT (400 MHz, D_2O): δ (ppm) 3.80–4.32 (broad, 6H, -NH_3), 4.89–4.94 (q, 2H, -CH_2-), 7.09–7.10 (d, 2H, py-H), 7.65–7.70 (m, 12H, Ph-H), 7.87–7.90 (m, 3H, Ph-H), 8.50–8.51 (d, 2H, py-H). ^{31}P-NMR (202 MHz, DMSO-d^6): δ (ppm) 23.15. ^{195}Pt-NMR (107 MHz, DMSO-d^6): δ (ppm) –2278.52. ESI-MS (positive mode, m/z) found (calcd): 307.33–312.33 (309.06), [Pt(*p*-PPh_3CH_2Py)$(NH_3)_2Cl$]$^{2+}$; 615.08–622.33 (619.01), [Pt(*p*-PPh_3CH_2Py)$(NH_3)_2Cl$−H]$^{+}$. Purity (HPLC): 97% (retention time: 2.38 min, eluent: CH_3OH/H_2O = 7/3).

2.5 Antitumor activity

The cytotoxicity of OPT, MPT, PPT, pyriplatin, and cisplatin at 48 h was tested by the MTT assay as we described previously.[3] Lung cancer xenografts were set up using STOCK-Foxn1^{nu}/Nju nude mice (5-week-old, female, n = 20). A549 cells (10^6) were inoculated subcutaneously in the back of mice. The mice were randomized into four groups when the tumors grew to 150–200 mm^3. One group was injected with different concentrations of OPT to optimize the dose; the rest were intravenously injected by saline, cisplatin, OPT (5 mg kg^{-1}) every other day for 19 days, respectively. The volume of tumor was monitored by a vernier caliper and calculated according to the following formula: Volume = ($width^2$ × length)/2. The body weight of mice was measured at the same time.

The biodistribution of the Pt^{II} complexes in mice was evaluated by ICP-MS after treatment for 19 days. Major organs as well as tumor tissue of mice were collected and weighed. The content of Pt was represented as ng Pt per gram of tissue. All the experimental procedures related to animals were performed in accordance with the

Guidelines for Care and Use of Laboratory Animals of Nanjing University and experiments were approved by the Animal Ethics Committee of the Model Animal Research Center of Nanjing University.

2.6 Cellular uptake

A549 cells (10^6) were treated with OPT, MPT, PPT and cisplatin (10 μM), respectively, for 24 h. The cells were collected and mitochondria isolation kit was used to separate the nuclei, mitochondrion-free cytoplasm and mitochondria. Samples were prepared as we described previously.[4] Pt content was determined by ICP-MS.

2.7 DNA binding

A549 cells (10^6) were treated with OPT, MPT, PPT, pyriplatin, and cisplatin (10 μM), respectively, for 24 h. Nuclei were isolated from cells by nuclei isolation kit, and nDNA was purified by TIANamp Genomic DNA kit. The content of nDNA-bound Pt was measured by ICP-MS. DNA concentration was determined by Nanodrop. Three parallel experiments were repeat.

A549 cells (10^6) were treated with OPT, MPT, PPT, and cisplatin (10 μM), respectively, for 24 h. Mitochondria were isolated from cells by mitochondria isolation kit, and mtDNA was purified by TIANamp Genomic DNA kit. Quality and quantity of mtDNA were determined as reported previously.[5] The damage of mtDNA was quantified using the $2^{-\Delta\Delta Cq}$ method, that is:

$$\text{Lesion rate [lesion per 10 kb DNA} = \left(1 - 2^{-(\Delta\text{long}-\Delta\text{short})}\right) \times \frac{10000[\text{bp}]}{\text{size } of\ long\ fragment[bp]}$$

Cell-free DNA binding was examined by 1% agarose gel electrophoresis in TAE running buffer (40 mM Tris acetate/1 mM EDTA, pH 7.4) on pUC19 plasmid DNA for 2 h at 90 V. Specifically, different concentrations of each complex were incubated with pUC19 plasmid DNA (200 ng μL^{-1}, 10 μL) at 37 °C for 24 h in Tris-HCl buffer (50 mM Tris, 50 mM NaCl, pH 7.4). OPT: 0, 20, 40, 60, 80, 100, 120, 140 μM, respectively; MPT: 0, 20, 40, 60, 80, 100, 120, 140 μM, respectively; PPT: 0, 10, 20, 30, 40, 50, 60, 70 μM, respectively. The total volume was 10 μL. The final concentration of pUC19 plasmid DNA is 20 ng μL^{-1}, the molecular weight of pUC19 plasmid DNA is 1.74×10^6 Da, 2686 base pairs. The concentration of nucleotide was calculated to be 61.75 μM. Formal drug-to-nucleotide ratios (r_f) are ranged from 0.16 to 2.24. The gels were stained with ethidium bromide (EB, 1 μg mL^{-1}) and visualized

under UVP gel imaging system. Unbound Pt was removed by spin microdialysis and bound Pt was determined by ICP-MS. DNA concentration was determined by Nanodrop. The bound drug-to-nucleotide ratios (r_b) were calculated. Three parallel experiments were repeat.

2.8 Mitochondrial respiration rate

The cellular respiration rate was measured with XF24 cell mito-stress test kit (Seahorse Bioscience, Billerica, MA).[6] A549 cells (10^4) were seeded in 24-well plates. The cells were treated with OPT, MPT, PPT, and cisplatin (10 μM), respectively, for 24 h. Prior to the assay, cells were supplemented with pyruvate (1 mM) and glucose (12 mM) as substrates to establish the basal oxygen consumption rate (OCR). Inhibitors of mitochondrial respiratory chain, including oligomycin (1 μM, ATP synthase inhibitor), carbonyl cyanide-p-trifluoromethoxyphenylhydrazone (FCCP, 1 μM, ATP synthesis uncoupler), rotenone (1 μM, complex I inhibitor) and antimycin A (1 μM, complex III inhibitor), were added sequentially to assess the changes of OCR using the XF24 Extracellular Flux Analyzer. Concentrations of protein were determined by Bradford assay kit.

2.9 Coupling assay of isolated mitochondria

Muscles of wildtype C57BL/6 mice (8 weeks, female) were surgically removed from anesthetized animals. Mitochondria from muscles were isolated according to MuCyteTM mitochondrial isolatation kit.[7] Mitochondrial protein was quantitated using Bradford assay kit. OPT and cisplatin (10 μM) were incubated with isolated mitochondria (4 μg) for 30 min before measurement respectively. The final concentrations of injections were 4 mM ADP, 1 μM oligomycin, 2 μM FCCP and 0.25 μM antimycin A, respectively. The changes of OCR were assessed using the XF24 Extracellular Flux Analyzer. The protocol was approved by the Institutional Animal Care and Use Committee of the Model Animal Research Center of Nanjing University.

2.10 Cellular citrate

A549 cells (10^6) were treated with OPT, MPT, PPT, and cisplatin (10 μM), respectively, for 24 h. Cellular citrate was determined by the citrate assay kit. The concentration of citrate (C_c) was calculated as follows:

$$C_c = S_a/S_v/50\ \mu L \times 191.2\ g\ mol^{-1}$$

where S_a is the amount of citrate in unknown sample (nmole) obtained from standard curve, S_v is the sample volume (μL) added into the wells.

2.11 Dissipation of MMP

A549 cells were treated with OPT, MPT, PPT, and cisplatin (10 μM), respectively, for 12 or 24 h, and carbonyl cyanide *m*-chlorophenyl hydrazine (CCCP, 50 nM) was used as a positive control. JC-1 mitochondrial membrane potential assay kit was used to stain mitochondria and the image was observed by confocal fluorescence microscopy (λ_{ex} = 488 nm). The red-to-green fluorescence ratio (R/G) was calculated with the Zone software.

2.12 Mitochondrial morphology

A549 cells (10^6) were treated with OPT, MPT, PPT, and cisplatin (10 μM), respectively, for 24 h. The cell samples were fixed and stained with uranyl acetate and lead citrate, and collected on copper grids. TEM images were taken on the JEOL JEM-2100 transmission electron microscope.

2.13 Release of cytochrome c

A549 cells (10^6) were treated with OPT, MPT, PPT, and cisplatin (10 μM), respectively, for 24 h. Mitochondria and cytoplasm of cells were separated by the mitochondrion isolation kit. Protein samples (90 μg) were separated on SDS-polyacrylamide gel electrophoresis, with 5% and 12% polyacrylamide for concentration and isolation, respectively. β-Actin mouse monoclonal-antibody (1:5000), Cyto *c* rabbit monoclonal-antibody (1:1000), goat anti-mouse IgG H&L (HRP) and goat anti-rabbit IgG H&L (HRP, 1:5000) were used to perform the western blot assay.

2.14 Apoptosis

A549 cells (10^6) were treated with OPT, MPT, PPT and cisplatin (10 μM), respectively, for 24 h. The cell samples were prepared using the BD Annexin V-FITC assay kit. The cells were quantitatively detected by flow cytometric assay after Annexin V-FITC and propidium iodide (PI) staining. Data were analyzed by the Flowjo 7.6.1 software.

2.15 Statistical analyses

Two-way Analysis of Variance (ANOVA) was used to ascertain significant differences between the controls and experimental data in the GraphPad Primer 7.00 software. Data were expressed as means ± S.D. (standard deviation).

3. References

(1) J. D. Buynak, A. S. Rao, G. P. Ford, C. Carver, G. Adam, B. Geng, B. Bachmann, S. Shobassy and S. Lackey, *J. Med. Chem.*, 1997, **40**, 3423–3433.

(2) S. N. Wu, X. Y. Wang, Y. F. He, Z. Z. Zhu, C. C. Zhu and Z. J. Guo, *J. Inorg. Biochem.*, 2014, **139**, 77–84.

(3) Z. Z. Zhu, X. Y. Wang, T. J. Li, S. Aime, P. J. Sadler and Z. J. Guo, *Angew. Chem. Int. Ed.*, 2014, **53**, 13225–13228.

(4) Z. Q. Zhang, Z. Z. Zhu, C. Luo, C. C. Zhu, C. L. Zhang, Z. J. Guo, X. Y. Wang, *Inorg. Chem.*, 2018, **57**, 3315–3322.

(5) O. Rothfuss, T. Gasser and N. Patenge, *Nucleic Acids Res.*, 2010, **38**, e24.

(6) D.-F. Wang, W.-T. Rong, Y. Lu, J. Hou, S.-S. Qi, Q. Xiao, J. Zhang, J. You, S.-Q. Yu and Q. Xu, *Acs Appl. Mater. Interfaces*, 2015, **7**, 3888–3901.

(7) M. D. Brand and D. G. Nicholls, *Biochem. J.*, 2011, **435**, 297–312.
